# Intraoperative Evaluation of Brain-Tumor Microvascularization through MicroV IOUS: A Protocol for Image Acquisition and Analysis of Radiomic Features

**DOI:** 10.3390/cancers14215335

**Published:** 2022-10-29

**Authors:** Giuseppe Roberto Giammalva, Anna Viola, Rosario Maugeri, Kevin Giardina, Rina Di Bonaventura, Sofia Musso, Lara Brunasso, Santiago Cepeda, Giuseppe Maria Della Pepa, Alba Scerrati, Giorgio Mantovani, Gianluca Ferini, Rosa Maria Gerardi, Maria Angela Pino, Giuseppe Emmanuele Umana, Luca Denaro, Alessio Albanese, Domenico Gerardo Iacopino

**Affiliations:** 1 Neurosurgical Clinic, Post Graduate Residency Program in Neurologic Surgery, Department of Biomedicine Neurosciences and Advanced Diagnostics, School of Medicine, University of Palermo, 90127 Palermo, Italy; 2 Department of Radiation Oncology, REM Radioterapia srl, 95029 Viagrande, Italy; 3 Department of Neurosurgery, Fondazione Policlinico Universitario A. Gemelli IRCCS, Università Cattolica del Sacro Cuore, 00100 Rome, Italy; 4 Departamento de Neurocirugía, Hospital Universitario Río Hortega, 47012 Valladolid, Spain; 5 Department of Translational Medicine, University of Ferrara, 44121 Ferrara, Italy; 6 Department of Neurosurgery, Sant’Anna University Hospital of Ferrara, 44124 Ferrara, Italy; 7 Trauma Center, Gamma Knife Center, Department of Neurosurgery, Cannizzaro Hospital, 95126 Catania, Italy; 8 Academic Neurosurgery, Department of Neurosciences DNS, University of Padua, 35128 Padua, Italy

**Keywords:** IOUS, microvascularization, brain tumor, MicroV, protocol, radiomics

## Abstract

**Simple Summary:**

The following paper aims to delineate a standard protocol for the analysis of brain-tumor microvascularization through the implementation of the intraoperative microvascular Doppler (MicroV) technique and the standardized acquisition of intraoperative ultrasound (IOUS) images during brain-tumor surgery. This study takes advantage of the BraTIoUS international database (ClinicalTrials.gov Identifier: NCT05062772), which is an international collaborative database of brain tumor IOUS images where MicroV images are stored and retrieved along with B-Mode images in order to be further analyzed by collaborating institutions. The proposed protocol aims to collect standardized MicroV images of brain tumors in order to analyze radiomic features of brain-tumor microvascularization. The study of brain-tumor microvascularization is therefore useful for a deeper knowledge of tumor behavior that ultimately results in an on-going adaptation of the surgery and in the improvement of surgical outcomes.

**Abstract:**

Microvascular Doppler (MicroV) is a new-generation Doppler technique developed by Esaote (Esaote s.p.a., Genova, Italy), which is able to visualize small and low-flow vessels through a suppression of interfering signals. MicroV uses advanced filters that are able to differentiate tissue artifacts from low-speed blood flows; by exploiting the space–time coherence information, these filters can selectively suppress tissue components, preserving the signal coming from the microvascular flow. This technique is clinically applied to the study of the vascularization of parenchymatous lesions, often with better diagnostic accuracy than color/power Doppler techniques. The aim of this paper is to develop a reproducible protocol for the recording and collection of MicroV intraoperative ultrasound images by the use of a capable intraoperative ultrasound machine and post-processing aimed at evaluation of brain-tumor microvascularization through the analysis of radiomic features. The proposed protocol has been internally validated on eight patients and will be firstly applied to patients affected by WHO grade IV astrocytoma (glioblastoma—GBM) candidates for craniotomy and lesion removal. In a further stage, it will be generally applied to patients with primary or metastatic brain tumors. IOUS is performed before durotomy. Tumor microvascularization is evaluated using the MicroV Doppler technique and IOUS images are recorded, stored, and post-processed. IOUS images are remotely stored on the BraTIoUS database, which will promote international cooperation and multicentric analysis. Processed images and texture radiomic features are analyzed post-operatively using ImageJ, a free scientific image-analysis software based on the Sun-Java platform. Post-processing protocol is further described in-depth. The study of tumor microvascularization through advanced IOUS techniques such as MicroV could represent, in the future, a non-invasive and real-time method for intraoperative predictive evaluation of the tumor features. This evaluation could finally result in a deeper knowledge of brain-tumor behavior and in the on-going adaptation of the surgery with the improvement of surgical outcomes.

## 1. Introduction

Brain tumors represents one of the most challenging CNS diseases in term of both diagnosis and therapy. In most of cases, surgery represents the crucial step of a patient’s care, even in association with chemotherapy and radiotherapy, for achieving the best outcome in terms of quality of life (QoL) and overall survival (OS) [1,2].

Neurosurgery has taken advantage of many technologies aimed at improving the surgical management of brain tumors. Beside the advancements in pre-operative diagnostic techniques, the need for the surgeon to have reliable intra-operative imaging support is still urgent; in fact, pre-operative tools such as neuronavigation and virtual/mixed reality are limited by surgical manipulation and brain shift [3]. This limitation may be overcome by the use of intraoperative imaging, such as intraoperative MRI, which suffers from high costs and logistic concerns, and intraoperative ultrasound (IOUS), which is already adopted in other surgeries and it can be performed several times during the procedure [4,5,6].

In recent decades, there has been increasing interest in IOUS in neurosurgery. In fact, IOUS allows a complete intraoperative anatomical and functional evaluation of brain tumors through the implementation of different techniques such as Doppler and contrast-enhanced ultrasound (CEUS) aimed at tailoring the surgical procedure according to intraoperative findings. Moreover, IOUS is easily repeatable several times and allows for evaluation of the residual mass, thus maximizing the extent of resection (EOR) of brain tumors [7,8,9,10,11,12].

In recent years, Doppler analysis has been improved with the introduction of a 3rd generation technology, which is more sensitive to little and low-flow vessels thanks to a suppression of interfering signals [13,14,15]. This technology has been clinically applied for the study of the vascularization of solid tumors from different organs, often with a better diagnostic accuracy than color Doppler/power Doppler techniques [14,16,17,18,19,20,21,22,23]. This technology is branded with different names according to the different producers and Esaote (Esaote s.p.a., Genova, Italy) has named its technology MicroV [13,14,15].

Since the wide use of IOUS in neurosurgery and the implementation of MicroV technology in ultrasound machines, MicroV can be effectively used intraoperatively for the study of tumor microvascularization even in brain-tumor surgery. In fact, important information about tumor histology, grading, and vascularization can be retrieved from the radiomic analysis of intraoperative MicroV images and the further translation of obtained data into the operative setting during surgery.

The objective of this study is to delineate a standard protocol for intraoperative MicroV image acquisition during brain-tumor surgery, their post-processing and first-order radiomics analysis. This protocol would be adopted systematically for the analysis of brain-tumor microvascularization, in order to implement the MicroV technique as an intraoperative diagnostic tool for brain tumors. The primary objective of this protocol is to allow quantitative analysis of qualitative data obtained by MicroV IOUS through radiomic analysis. The analysis of MicroV radiomic features would enhance the knowledge about tumor histotype and behavior. Thus, results obtained from this data can be exploited during surgery in order to improve surgical outcomes, through the study of tumor microvascularization according to different histologies, the intraoperative detection of misdiagnosed intratumoral progression, and the better definition of tumor boundaries.

## 2. Materials and Methods

### 2.1. Study Design

This study protocol was developed by our institution (Unit of Neurosurgery, A.O.U.P. “Paolo Giaccone”, University of Palermo, Palermo, Italy) in collaboration with the Department of Neurosurgery, Fondazione Policlinico Universitario A. Gemelli IRCCS (Rome, Italy) since the same IOUS devices and technology were adopted in both institutions. Multicentric collection, database storage, and analysis of IOUS images of patients who had undergone brain-tumor surgery had ethical approval (protocol no.5/2022 Palermo 1 Ethical Committee). In addition, our institution actively collaborates with the BraTIoUS international database (ClinicalTrials.gov Identifier: NCT05062772).

IOUS MicroV images retrospectively recorded during the development of the present protocol and those which will be further prospectively obtained will be anonymized and stored along with B-mode images on the BraTIoUS database.

### 2.2. Patient and Public Involvement

Patients underwent this protocol in cases where informed consent was given to IOUS image acquisition and storage on the international database. Data obtained from MicroV image analysis were collected according to the present protocol and further analyzed. The results of data analysis will be focus of scientific investigations and will be published through academic conferences and international scientific journals.

### 2.3. Patient Recruitment

In the first stage of this protocol, the target population was represented by adult patients of any gender who were suspected of being affected by WHO grade IV astrocytoma (glioblastoma—GBM) according to pre-operative imaging, and who were candidates for craniotomy and lesion removal. In a later stage, patients with other primary or metastatic brain tumors will also be enrolled. Patients were enrolled by the participating institutions and were recruited only if the inclusion criteria were met at each participating center.

### 2.4. Eligibility Criteria

Inclusion criteria were: (1) patients affected from a brain tumor undergoing craniotomy and lesion removal; (2) obtained informed consent for intraoperative ultrasound; (3) obtained consent for IOUS image acquisition and storage on international database.

Exclusion criteria were: (1) no informed consent for IOUS; (2) no informed consent for IOUS image acquisition and storage on international database; (3) craniotomy not suitable for IOUS or smaller than probe width along longitudinal field of the tumor; (4) craniotomy not sufficient for the correct placement of the probe; (5) history of former cerebral surgery affecting IOUS acquisition; (6) relapsing brain tumor.

### 2.5. IOUS Devices and Set-Up

A last generation ultrasound machine (Esaote MyLab Twice, Esaote s.p.a., Genova, Italy) equipped with software to perform MicroV analysis was adopted to acquire IOUS images with the MicroV technique. The ultrasound machine was provided by a linear 3–11 MHz multifrequency probe (LA332, Esaote s.p.a., Genova, Italy).

Standard parameters to be set on Esaote MyLab Twice machine at the beginning of the IOUS acquisition are listed below (Table 1).

### 2.6. Protocol of IOUS Image Acquisition

The operating room was large enough to allow the positioning of the ultrasound machine next to the first surgeon and to allow the surgeon to freely access the user interface. Touch monitor and keyboard were covered with transparent sterile drape. The transducer was covered by sterile ultrasound gel and the probe wrapped in a transparent and sterile sheath.

All the images were acquired after the craniotomy was completed and before the durotomy. The craniotomy should be wide enough to allow the probe placement (minimum diameter 3.5 cm) and to allow probe free orientation and tilting. During the IOUS acquisition, the surgical field was filled with saline solution in order to obtain a better acoustic coupling.

#### 2.6.1. B-Mode

First, the tumor was identified in B-mode. IOUS was performed in order to visualize the tumor along its largest diameter (longitudinal field); the purpose is to find a useful plane to visualize a single image of the tumor including as much volume as possible.

In cases of mixed necrotic and solid areas, the useful plane should limit the presence of necrotic zones and should allow the greatest possible visualization of representative solid areas of the tumor. Once the best useful plane has been identified, it is mandatory not to move the probe or change the US plane. At this point, a first picture was acquired.

#### 2.6.2. Color Doppler Mode

In the next step, B-Mode was switched to color Doppler mode.

After enabling the color box, the box had to be adjusted so that the size was approximately 50% of the surface of the tumor and it was included exclusively within the tumor, avoiding the extension beyond the tumor borders as visualized in B-mode.

In cases of necrotic areas, the box should be placed on a region where the solid part of the tumor is most represented. If necessary, reduce the size of the box to below 50%, just enough to exclude any necrotic areas.

In order to refine the image, color gain should be increased until the appearance of acoustic artifacts; then, it has to be progressively reduced and set as soon as artifacts disappear. At this point, another picture was acquired.

#### 2.6.3. MicroV Mode

Paying attention to not moving either the color box or the probe, MicroV modality is activated by pressing the button on the touchscreen of Esaote MyLab Twice.

In order to refine the image, even at this stage color gain should be increased until the appearance of acoustic artifacts, then it has to be progressively reduced and set as soon as artifacts disappear.

Since MicroV has high sensitivity to low echoes, surgeon should wait a few seconds with the probe perfectly still for the complete reception of the echoes and the generation of the image. At this point, another picture was acquired and then it was possible to disable MicroV mode.

#### 2.6.4. Image Acquisition of the Tumor Periphery

Back to Color Doppler mode without moving the probe, the color box should be moved straddling the periphery of the lesion, in an area previously identified as hypervascularized and not necrotic.

Again, in order to refine the image color, gain should be increased until the appearance of acoustic artifacts; then, it has to be progressively reduced and set as soon as artifacts disappear. At this point, another picture was acquired.

Without moving the box, MicroV mode was selected as described before and color gain should be increased until the appearance of acoustic artifacts; then, color gain has to be progressively reduced and set as soon as artifacts disappear. After a few seconds with the probe perfectly still for the complete reception of the echoes, another picture was acquired.

**Figure 1:**
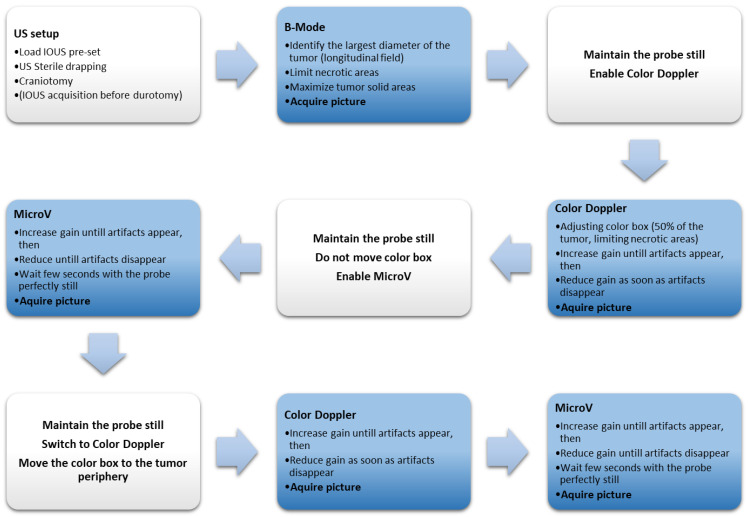
Step by step flowchart of the standardized protocol of IOUS image acquisition.

If correctly executed, the ultrasound plane and tumor visualization do not change during the entire execution of the protocol (Figure 1).

### 2.7. Protocol of Post-Acquisition Analysis

Once all the pictures had been acquired, they were exported in a DICOM format, used for post-processing and first-order radiomics analysis of texture features.

Post-processing, preparation of images, and first-order radiomic analysis were performed with ImageJ v.1.53 (*Wayne Rasband, National Institutes of Health, Bethesda, MD, USA*), an open-source Sun-Java-based image processing software used for scientific image analysis [24].

First, DICOM images were individually imported into ImageJ using the Bio-Formats Importer plugin (*Bio-Formats, Open Microscopy Environment, University of Dundee, Dundee, UK*) with the following parameters: color mode = Default; view = Hyperstack; stack order = XYCZT.

The following stages of image post-processing were different according to whether MicroV images have been acquired with *MV0 color map* (which is the standard pre-set color scale, resulting in a color-coded map where different colors corresponding to different flow rates) or with *MV2 color map* (which results in a 256-tone greyscale map where the total white corresponds to a value of 255 and represents the highest flow rate; whereas the total black corresponds to a value of 0 and represents the absence of flow).

#### 2.7.1. Post-Processing of MicroV Images

If MicroV images were acquired with *MV0 color map*, they would result in color images and they needed to be converted into grayscale in order to be further analyzed.

After the importation with Bio-Formats Importer plugin, the stacked image was converted in RGB color mode. Then, the RGB color image was retro-converted into an 8-bit image with 256 shades of gray (255-0) using a custom Look Up Table (LUT) built on the basis of Esaote MicroV MV0 color scale (Supplementary Materials).

The custom LUT was calculated by dividing the color scale displayed on the MicroV Image in 256 segments. Each segment corresponds to a precise RGB color; a progressive value from 255 to 0 was assigned to each color of the MicroV color scale, which corresponds to a progressively increasing gray intensity (255 white, 0 black) and consequently to a different value of blood flow. According to the custom LUT, the value 255 was assigned to the first color of the chromatic scale (which was then converted to white and corresponds to the highest blood flow) while the value 0 was assigned to the last color of the chromatic scale (which was then converted to black and corresponds to the absence of blood flow). According to the segmentation in 256 color segments, a progressively decreasing value was assigned to each color segment on the chromatic scale in order to convert colors in shades of gray with progressively lower intensity value according to the flow rate.

**Figure 2:**
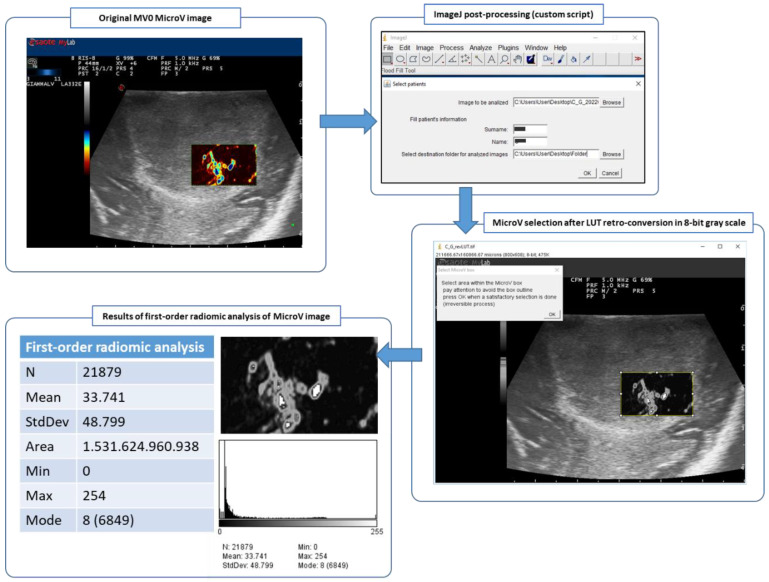
Post-processing ImageJ workflow of MicroV IOUS image with MV0 color map, from the original image to the 256 tones 8-bit grayscale image and finally to the first-order radiomic analysis of the selected area within the MicroV box.

This means that the color-coding related to the flow rates was converted to grayscale, whereby higher flow rates corresponded to higher values of the grayscale and were converted to whiter pixels whereas lower flow rates corresponded to lower values of the grayscale and were converted to blacker pixels (Figure 2).

Even if this retro-conversion was developed on an MV0 color-map for the purpose of this protocol, different custom LUTs could be calculated for different color-maps or US machines simply by following this procedure.

This retro-conversion was made by custom Java script for ImageJ (UndoLUT.ijm), which relies on the custom LUT just calculated and which has been freely adapted for the purpose of this protocol (Supplementary Materials) [25]; this generated a final 8-bit grayscale image, which was then saved in TIFF format.

If MicroV images have been acquired with an MV2 color map, they already result in 256-tone grayscale images and do not require further retro-conversion. According to this color map, different flow rates correspond to different shades of gray: the highest flow rate corresponds to a value of 255 and it is depicted total white, whereas the absence of flow corresponds to a value of 0 and it is depicted total black.

#### 2.7.2. First-Order Radiomic Analysis

With an 8-bit grayscale image, the area of the MicroV box was evidenced using a rectangular selection, paying attention to excluding the outline of the box itself.

With intrinsic analyzing tools of ImageJ, the following information were calculated on the selected area: *selected area; average, minimum and maximum intensity value* of selected pixels; and *standard deviation* and *histogram* with quantitative distribution of pixels according to their intensity. In this way, higher values corresponded to faster and more-represented flow rates, whereas lower values corresponded to slower and less-represented flow rates. Distribution of pixels per intensity might represent the pattern of microvascularization.

Numerical data thus obtained were recorded for further analysis and the histogram of pixel distribution were saved in TIFF format (Figure 2).

For the purpose of this protocol, comprehensive Java scripts for ImageJ were developed in order to guide the entire post-processing for both MV0 and MV2 color-map images. More information about UndoLUT.ijm source code for LUT retro-conversion in ImageJ and the final ImageJ Java scripts adapted and developed by the authors for the purpose of this protocol may be found in the Supplementary Materials [25].

### 2.8. Data Collection and Management

All anonymized IOUS images were stored on the BraTIoUS database and anonymized data on patients’ demographics, clinical variables, sonographic characteristics, and post-acquisition analysis were collected and stored in a digital data bank accessible to each referring physician involved in this study. At the end of the acquisition, data collected according to this protocol will be available to participating institutions for further radiomic analysis regarding intratumoral and peritumoral features of brain tumors, the differentiation from the surrounding brain, and the characteristic differences among several histotypes. Final results will be published in an anonymized form.

## 3. Results

The primary purpose of this paper is to delineate a standardized protocol of acquisition and analysis of intraoperative ultrasound images using the MicroV Doppler technique.

The secondary purpose is to implement the diagnostic capabilities of IOUS with the standardized use of MicroV. In particular, through this standardized protocol of MicroV image acquisition and post-processing, we aim to promote radiomic analysis of brain-tumor microvascularization in order to improve intraoperative diagnosis and to guide the further surgical and therapeutic course.

### Protocol Refinement and First Proof-of-Concept Applications

In order to perform an internal validation, this protocol has been refined on eight patients affected by WHO grade IV astrocytoma (GBM) who underwent craniotomy and removal of brain tumor at A.O.U.P. “Paolo Giaccone”, University of Palermo (Palermo, Italy) and Fondazione Policlinico Universitario A. Gemelli IRCCS (Rome, Italy). After proper informed consent for IOUS and anonymized image-publishing on the international database was collected, each patient underwent craniotomy for lesion removal. IOUS was performed after craniotomy and before durotomy and ultrasound images were recorded according to the above-defined protocol.

Patients were three males and five females, mean age 62.25 years (range 45–71 years); three were affected by frontal GBM (two right, one left), two by temporal GBM (one right, one left), one by right parietal GBM, another by IV ventricle GBM, and the last one by left temporal–parietal–occipital GBM. Each patient underwent gross-total resection (GTR) of the brain tumor.

**Figure 3:**
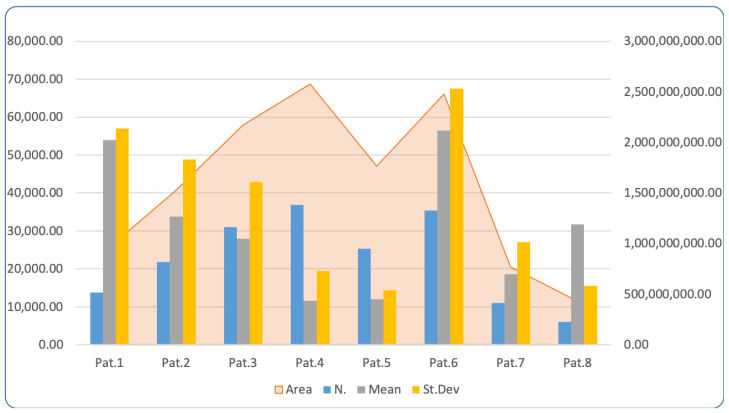
Graphic data from first-order radiomic analysis performed with ImageJ on the first eight GBM patients as specified in Table 2. Area in expressed in px^2^ on secondary Y-axis.

**Figure 4:**
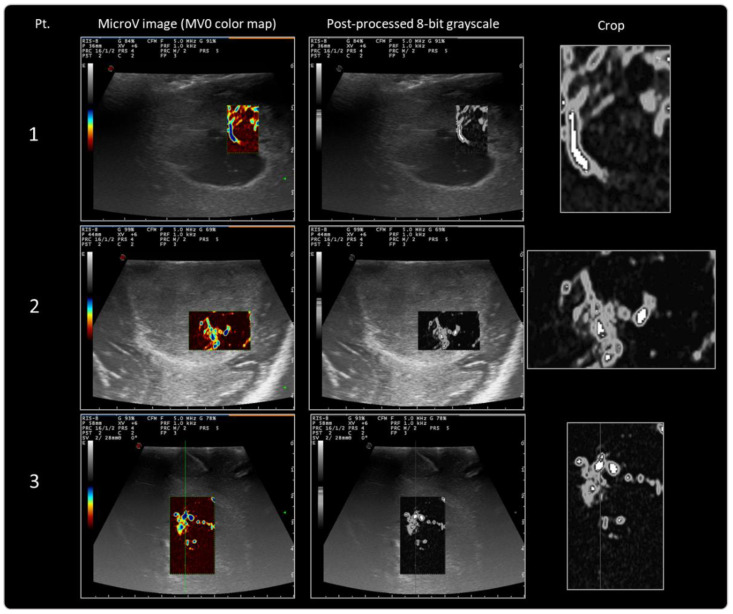
Results of MicroV IOUS images with MV0 color map post-processing for internal validation of the protocol.

MicroV images which were acquired during IOUS were further post-processed according to the above-defined protocol. Data obtained after post-processing and first-order radiomic analysis are reported in Table 2 and Figure 3, and MicroV images thus processed are ready for further more advanced radiomic analysis (Figure 4). First-order radiomic analysis lies in texture analysis, which quantitatively analyzes how intensity features are spatially distributed inside the MicroV images. Average, minimum, and maximum intensity values, standard deviation, and a histogram with quantitative distribution of pixels within the selected area were calculated on the first eight GBM patients enrolled. Texture analysis represents the first step toward subsequent higher-order analysis, which requires a higher number of enrolled patients and available images.

## 4. Discussion

Pertinent literature from the last few years focuses on overcoming the pitfalls of even the most innovative tools used for brain-tumor surgery. It is known, for example, that 5-aminolevulinic acid (5-ALA) has shown a major improvement in the identification of pathological tissue at high-grade glioma margins, in contrasting the inadvertent residuals after gross total resection, and then in both overall and progression-free survival; despite this, it was demonstrated that 5-ALA can also fail to identify some pathological areas when there are technical limitations to tumor visualization based on microscopic light, orientation, or craniotomy features. Contrast-enhanced ultrasound-assisted techniques demonstrated high impact in integrated intraoperative protocol [26].

In this progressive scenario, MicroV Doppler is able to overcome the limitations of conventional Doppler techniques by an adaptive algorithm. This is capable of filtering clutter artifacts by the suppression of low-frequency components derived from the patient’s movements and breathing and by the preservation of extremely low-flow signals in order to enhance microvasculature. In particular, MicroV uses advanced filters, which can differentiate tissue artifacts from low-speed blood flows; by exploiting spatial–temporal coherence information, these filters can selectively suppress tissue components, preserving, at the same time, the signal coming from the microvascular flow. The flow is shown through either an overlaying colored image or a grayscale image with subtraction of the underlying B-mode image [13,14,15].

According to the most recent, yet scarce, literature on this matter, MicroV has been shown to be more sensitive than traditional Doppler methods in the study of small hepatocarcinoma (<2 cm) [17], as well as in the evaluation of benignity or malignancy of cervical lymph nodes [18], in the differential diagnosis of suspicious renal lesions [19] and for the evaluation of breast-cancer vascularization [20]. Beyond oncological applications MicroV sensitivity and effectiveness have been also assessed in the evaluation of carpal tunnel syndrome [21] and testicular vascularization [14,22]. Regarding neurological and neurosurgical applications, MicroV has been effectively used for the evaluation of brain perfusion in newborns and for the study of Schwannomas microvascularization [14,23]. Recently, another application of microvascular doppler called the superb microvascular imaging (SMI) technique has been combined with US monitoring during brain-tumor surgery and biopsies, showing advantages in improving recognition of the tumor margin, in providing a clear visualization of normal anatomic morphology of the normal brain zones surrounding the tumor and the vessels, and in overcoming the problem of brain shifting in intraoperative neuronavigation [27]. SMI has been subsequently implemented with contrast-enhancement, in a series of twenty patients diagnosed with brain tumors; this association has proved to be effective in visualizing tumor borders by the difference between tumor flow and brain flow, by the contrast between increased density of tumor vessels and decreased appearance time of the contrast agent compared to normal brain vessels [28].

Due to this early evidence and the easy implementation of MicroV during IOUS, we propose a standardized protocol of MicroV image acquisition during IOUS for brain-tumor surgery, image post-processing, and fist-order radiomic analysis. The aim is to foster both the systematic implementation of MicroV acquisition and to promote further radiomic analysis of brain-tumor microvascularization on the basis of data retrieved from these images. Moreover, data obtained from several images and the further radiomic analysis of texture features would be beneficial both during and after surgery. In fact, a deeper knowledge about brain-tumor microvascularization obtained from the analysis of MicroV images radiomic features would both improve surgery by direct intraoperative assessment of the tumor and by the post hoc analysis of tumor behavior and histotype according to radiomic data obtained earlier.

In this way, qualitative information can be quantitatively analyzed, and several outcomes would be possible: to find a correlation between the pattern of microvascularization and lesion histology, to enhance the predictivity of pre-operative diagnostics, to differentiate intraoperatively between high-grade and low-grade tumors, and to identify primitive from metastatic lesions. From an operative point-of-view, real-time intraoperative information can be acquired by the surgeon and integrated to other pre-operative and intraoperative tools in order to tailor the surgery. This could be obtained by better defining tumor boundaries and resection margins, by intraoperatively defining the presence of misdiagnosed intratumoral high-grade focal progression of low-grade gliomas, and by evaluating the relationship between intraoperative fluorescence patterns and tumor microvascularization in order to finally improve the patient’s outcome [29,30,31,32,33].

The standard acquisition of MicroV images and their radiomic analysis could improve patient’s outcome, reducing post-operative complications, increasing of the overall survival and ensuring the best onco-functional balance achievable. According to this perspective, the more cases are collected in such a standardized way the greater the possibilities of data analysis; in this regard, storage of cases and IOUS images on a remote and multicentric database is extremely helpful in widening and differentiating the collection of MicroV images, thus improving radiomic analysis and reaching more consistent significance. Thus, we also welcome additional centers that are already participating in the BraTIoUS database and other centers meeting relevant standards to join in applying this protocol.

This protocol has certain limitations: its application depends on the availability of a dedicated ultrasound machine equipped by MicroV software, the reliability of IOUS is characteristically operator-dependent, the internal validation of this protocol is still limited to eight cases, and the protocol has been applied and validated on MV0 color-map MicroV images. However, despite the first limitation being restrictive, the other limitations could be overcome just by the strict application of the protocol and the consistent collection of cases which will allow a stronger internal and external validation. Moreover, thanks to the possibility of calculating custom LUT for different color-maps than Esaote MV0, this protocol of acquisition and post-processing may be extended to other US machines capable of acquiring 3rd-generation Doppler for the study of microvascularization. In conclusion, this protocol would not be limited only to MicroV IOUS images but it could be suitable also for the acquisition and analysis of advanced IOUS images other than MicroV with appropriate adjustments.

## 5. Conclusions

MicroV represents a safe and effective IOUS technique which can be used intraoperatively for the study of tumor microvascularization in brain-tumor surgery.

In the proposed protocol for standardized intraoperative MicroV image acquisition during brain-tumor surgery, the post-processing and first-order radiomics analysis of texture features is easily reproducible and has already been internally validated on eight patients. This protocol aims also to foster the use of MicroV as an intraoperative diagnostic tool during brain-tumor surgery, thus improving knowledge about brain tumor characteristics and behavior by the analysis of their radiomic features.

## Figures and Tables

**Table 1:** Ultrasound parameters set for standardized MicroV IOUS image acquisition on Esaote MyLab Twice ultrasound machine according to the proposed protocol.

B-Mode	
Depth	44 mm
Gain	85%
Power	80%
Mechanical index	0.4
Probe frequency	6–8 MHz (tissue-enhancement imaging mode: low resolution)
Dynamic range/sharpness/density	16/1/2
PRS (persistence)	4
Post-processing	2
XView	+6
**Color (to be set in addition to former B-mode parameters)**	
Mechanical index	0.2
Color frequency	5 MHz
Color PRF	1.0 KHz
Color gain	50%
Color PRC	M/2
Color PRS (persistency)	6
**Doppler (to be set in addition to former Color mode parameters)**	
Mechanical index	0.3
SV	3/22 mmθ
Doppler Frequency	4.2 MHz
Doppler PRF	3.0 KHz
Doppler PRC	6
**MicroV Doppler (to be set in addition to former Doppler mode parameters)**	
Color PRS (persistency)	5
HD-CFM	2
MAP COLOR	MV0 or alternatively MV2
DIM-TP	56°
SENS	5
SMOOTH	Medium
Clip duration	Unlimited
Filter	3
Plane	0

**Table 2:** Data from first-order radiomic analysis performed with ImageJ on the first eight GBM patients according to and for the refinement of the proposed protocol. *Pt*: patient, sex, and age; *N*.: number of pixels analyzed; *Area*: area of the rectangular selection for analysis; *Mean*, *StDev*, *Min*, *Max*, *Mode*: these data refer to the intensity value from 255 to 0 of each pixel within the selected area on 8-b gray images according to gray tone.

Pt.	Histology	Location	N.	Area (px^2^)	Mean	StDev	Min	Max	Mode
F, 68 y	GBM	Lt. frontal	13,775	964.309.787.326	53.884	56.996	0	254	8 (645)
M, 57 y	GBM	Rt. frontal	21,879	1.531.624.960.938	33.741	48.799	0	254	8 (6849)
M, 71 y	GBM	Lt. temporo-parieto-occipital	30,989	2.169.364.500.868	27.887	42.783	0	254	8 (3651)
F, 66 y	GBM	Rt. parietal	36,816	2.577.279.791.667	11.529	19.424	0	190	8 (32676)
F, 62 y	GBM	Rt. frontal	25,252	1.767.749.600.694	12.075	14.387	0	174	8 (10259)
M, 67 y	GBM	Rt. temporal	35,412	2.478.993.697.917	56.478	67.567	0	254	254 (2644)
F, 45 y	GBM	IV ventricle	10,998	769.907.734.375	18.649	27.067	0	254	8 (3617)
F, 62 y	GBM	Lt. temporal	6102	427.166.484.375	31.701	15.528	0	180	28 (401)

## Data Availability

The data presented in this study (Custom MV0 LUT table, custom ImageJ Java script for MV0 image post-processing, and custom ImageJ Java script for MV2 image post-processing) are openly available on Zenodo.org at https://doi.org/10.5281/zenodo.6849562, reference number [25] Source code of UndoLUT.ijm custom Java script for LUT retro-conversion may be found at https://github.com/chalkie666/imagejMacros/blob/04818b8ed6805a0ab000ab815ed2fff7290b1aae/UndoLUT.ijm, accessed on 1 May 2022.

## Supplementary Materials

The following supporting information can be downloaded at: https://doi.org/10.5281/zenodo.6849562, accessed on 17 July 2022: MicroV_MV0.lut, custom MV0 LUT table; Analyze_Micro_v1.0 MV0.txt, custom ImageJ Java script for MV0 images post-processing; Analyze_Micro_v1.0 MV2.txt, custom ImageJ Java script for MV2 images post-processing.
